# 

**Published:** 1889-12-28

**Authors:** 


### SATURDAY, DECEMBER 28th, 1889.

**Epidemics and Ambulances** - 193
**Words of Consolation**—The Babe of Bethlehem - 194
**The Doctor's Pulpit.**—Christmas "Outward and Visible" 195
**Mr. Besant's Latest Morality** - 196
**The History and Capabilities of Herbal Simples.** By W. T. Fernie, M.D. - 197
**Notes from Scandinavia** - 198
**Old Cures for Hydrophobia** - 198
**Everybody's Page** - 199
**Notes and News** - 200
**Annotations.**—Dabblers in Electricity—Do Doctors Despise the Influenza Epidemic?—Hereditary Sleeplessness - 202
**The Hospital Workshop.** No. XIX.—St. Luke's Hospital for the Insane - 206
**The Practitioner's Outlook and Retrospect:**
Medicine—Winter Cough—Prevention of Cholera—Hay Fever - 203
Surgery—Clergyman's Sore Throat—Chronic Suppurative Pyelitis—Colotomy - 204
Recent Research—The Effect of the Entrance of Air into the Circulation - 205
Hygiene—Cycling in its Relation to Health - 205
Dietetics—Fish as Food - 205
**Influenza** - 207
**Cottage Hospital Ward Walls** - 207
**The Proposed Registration of Nurses** - 207
**Fallow Crops.**—A Hospital of all the Virtues - 208
**Scraps and Gleanings** - 208

**EXTRA SUPPLEMENT—THE NURSING MIRROR.**

**En Passant.**—Looking Back—Growing Old—District Nursing—Secession to Rome—Bolton Workhouse Scandal—Rural Nursing Association—Nurses' Home at Newcastle—Care of the Hands - liii

**Old Jeremy's Visitor** - liv

**Keeping Christmas** - lv

**Registration** - lv

**Answers to Examination Questions** - lv

**Everybody's Opinion.**—Asylum Attendants, lv; Overworked Nurses - lvi

**Death in Our Ranks—Lectures** - lvi

**Notes and Queries—Vacancies—Amusements** - lvi

